# Commentary: Physical time within human time

**DOI:** 10.3389/fpsyg.2023.1110386

**Published:** 2023-05-26

**Authors:** Mauro Dorato

**Affiliations:** Department of Philosophy, Communication and Performing Arts, Roma Tre University, Rome, Italy

**Keywords:** time reversal invariance, brain limitations, IGUS model, anticipation, temporal perspectives

I will start my comments to the two target papers [Gruber et al., 2022 (GBM) and Buonomano and Rovelli, 2022 (BR)] with two uncontroversial premises, stressed in particular by BR:

1. “The function of our brains is to *anticipate the future”* (Buonomano, 2017, 232).
2. We anticipate the future in the present by using *relevant inductive information* stored in our memory.
I take it that 1 and 2 are sufficient to conclude that:
C. Across time we experience three different temporal perspectives about the *same* physical events: *anticipation, perception, and memory*. Notice that these events need *not* be temporally close to our present experience: I can anticipate my giving a talk next month and then remember it for a long time.
If this argument is correct, a few crucial questions arise:
3.1. How can the *same* event be first anticipated in the non-immediate future, perceived, and then remembered in the past?
3.2. What is the ontological status of the anticipated events? Do they exist tenselessly in a block (a) *or* do they come into being when they occur (b)?
3.3. Is there a genuine difference between the alternatives (a) and (b)?

By referring to the two target papers, I will focus on the first two questions^1^ by briefly sketching three possible avenues of research: *physical, ontological*, and *neurocognitive*.

One *physically* necessary condition for C is that events “keep on happening” one after the other along worldlines. IGUSs rely on this presupposition too: our brains register the objective temporal succession of physical events, where the objectivity is given by the invariance of proper time. GBM agree: “the experience of happening is part of our experience of the flow of time” (p. 6). In BR, Rovelli insists that spacetime is replete with processes and therefore is “dynamic”.

The *ontological* way to “explain” or describe this succession of physical events is to postulate a “locally growing block (Ellis, 2014), where *local* is added to prevent objections raised by relativity”^2^. I claim that evidence for this model are ***facts*** that do not involve our momentary experience of time that IGUSs^3^ are meant to simulate but my *knowing* that, as I write at T, each *passing* day I am 1 day “closer” to the moment of my death D. Relatively to T, every minute the number of heartbeats separating T from D for me decreases in average by sixty: time for me passes in average one heartbeat per second!

The problem with the locally growing block is, as GBM correctly note, that it seems to be unable to shed light on the two-times problem from an *empirical* viewpoint. Yet, it is difficult to account for the facts above just by postulating a tenseless, “static” relation between T and D. I grant that this explanation can be given and that physics, obviously, does not require a privileged now. However, despite the following spatial metaphors, the claim that, relative to T, the *temporal distance* between T and D decreases seems much more plausible: this fact calls for a locally growing block regarded as a *primitive, fundamental ontological asymmetry* or as a “irreducible intrinsic asymmetry in the temporal structure of the universe” (Maudlin, 2007, p. 109).

A first difficulty is that, contrary to C, IGUSs work only for events that are closer to our present experience by including our short-term *memory*: as such, they do not seem capable to simulate the essentially predictive capacity of our brain (see 1 above). If anything, IGUSs can only refer to *short-term* anticipations (Dorato and Wittmann, 2020). Neither can “premonitions about the future” (p. 3) solve this problem.

More in general, the IGUSs presented by GBM seem too simple to account for the complexity of our experience of time. Evidence for this claim is that whenever some discrepancies between the IGUSs and our experience arise, the former must be supplemented with *additional* “gadgets” (GBM, p. 3). On the one hand, the simulation must be faithful. On the other, IGUSs cannot be too complex since this would imply providing them with too many contraptions^4^. Faithfulness and simplicity pull in opposite directions. In addition, the mere *possibility* to fabricate various IGUSs by using VR headsets to show that our experience of flow might be illusory does not imply that our *actual experience* is not veridical. If “the experiential flow component of the FOT is attributed to the utilizing system of the robot and not to the time of physics” (p. 2), the illusions that it generates are themselves “real” even if subjective, because the robot itself is *physical*. Furthermore, a thorny conceptual difficulty is generated by the widespread use in physics of the vague *epistemic* term “information” (which enters the definition of IGUSs): given that the notion contains a *semantic* feature that seems irreducible, what is information in physical terms? “To be informed that…” has a propositional content (a “that clause”) and propositions are *abstract*, non-physical entities.

GBM hold that FOT presupposes “dynamism of change/motion” (p. 4). The problem raised by this quotation depends on the meaning of ‘dynamism and change’. Correctly denying any motion of the now does not rule out some minimal form of tenseless becoming: the caption of Figure 1 tells us that “the robot experiences a stack of cards labeled a, b, c, d, e, f, whose top member *changes from time to time*” (GBM, p. 2). However, this sequence can more plausibly be interpreted as a worldline-dependent *coming into being* of events at instants of proper time, as suggested by the above argument concerning the decreasing distance between T and D. The anticipation of an event in the distant future and its later experience in the present presupposes some stronger kind of dynamism consisting in the addition of previously *non-existing* events in an *unrestricted sense of existence*^5^ that cannot be explained away by the momentary experience of flow allegedly allowed by the IGUSs.

Within neurophysiology, if 1 above presupposes a capacity for *mental time travel* (a projection in different moments of time) it also requires an *enduring* self. Mental time travel has been the subject of intense experimental study (Suddendorf et al., 2009). For instance, the use of spatial metaphors in our talking about time may depend on the fact that “Time Travel and Mental Space Navigation could be consistently explained by similar cognitive mapping principles, namely: egocentric mapping and coordinate system conversion” (Gauthier and van Wassenhove, 2016, p. 66). Egocentric mappings are representations of time (and space) from our temporal perspective (like the “here” in a map). Coordinate transformations are self-projections maintaining “egocentricity of the map when adopting a viewpoint differing from the ‘here and now”’ (Gauthier and van Wassenhove, 2016). It seems to follow that the possibility of keeping the egocentric character of the map entails an enduring self.

It has also been stressed that within a *subjective, agential* perspective, the self must be regarded as an *enduring entity* (Paul, 2017)^6^. The *agential* viewpoint implied in our temporal projections is directly called into play by 1: the capacity to predict a future event has been selected by evolution and serves the subject's need to *act* in view of an anticipated event.

I agree with BR that time is a multilayered concept. The list from i) to ix) (p. 5) is an inventory of key temporal notions apportioned between physical and neuropsychological time: in this respect, I argued that the main bridge between them is an *ontology of events* (Dorato, 2015). Since this plurality of senses holds even among the various branches of physics (Rovelli, 2004, p. 58–62), progress in the two times problem depends on disentangling the various elements in the list.

First, a radical pluralism about time and a “dappled view of science” in general (Duprè J., 1993; Cartwright, 1999) would dissolve our problem: within this framework we shouldn't even try to *reduce* or *unify* notions of time belonging to very different “levels of reality”. If physical time and experiential time have a limited, domain-relative range of validity, they cannot conflict. However, the two-times problem ought to be regarded as an attempt to bridge an *explanatory* gap and I take it that BR implicitly assumes that explanation need *not entail* reduction.

Second, reliance on the oft-invoked but unclear notion “*open future*” requires attention. BR identify “open” with “indeterministic”: “Neuroscience builds on the existence of macroscopic traces and on the openness of macroscopic future produced by the thermodynamic arrow of time. The second, in particular, underpins the possibility of our experience of being ‘free to choose’, since different macroscopic futures are compatible with the same macroscopic past, choice depends on what happens in the organism” (BR). Also for compatibilists choice “depends on the organism.” The existence of different possible futures all compatible with the same past requires *indeterminism* but the thermodynamical arrow of time depends on the initial state of the system. The fact that statistical mechanics is both *deterministic* and *time-symmetric* implies that the indeterminism in question is epistemic like the probabilities involved in the theory. In addition, the incompatibility between freedom and determinism is very controversial^7^.

Also the expression “four-dimensional block” must be handled with care. Analytical philosophers usually argue that the block, regarded as the sum total of events and changes in four dimensions, is static, since changes and events happen *in* the block. Rovelli correctly reminds us that general relativity implies a different account of the “block” because its main novelty is that *spacetime itself* (i.e., the block) *is dynamical:* “The 4-dimensional universe is not an entity, it is a process…. a complex network of changes, not a static 4-dimensional block”. I am sure that Rovelli agrees that in some sense the (observable) universe *is an entity* and that, unlike any other process, it does not occur *in* time, but has time as one of its dimensions.

Finally, Buonomano stresses the fact that the function of our brain, shaped by evolution (see 1 above) creates unavoidable limitations to the task of interpreting those physical theories referring to layers of reality that are very remote from our experience. He claims correctly that physics does not *force* us to adopt any particular temporal ontology and that, possibly as a consequence of the fact that our brain mainly relies on visualization, we cannot picture quantum jumps caused by photons hitting electrons in the nucleus. Yet, these limitations do not imply that realism about the ontological claims of physical theories is unjustified. The undeniable cognitive “inadequacies” of our brain are not a safe guide to ontology: both the discovery of inertia and of the relativity of simultaneity clearly show that the naïve physics implanted in our brain by evolution can be conquered.

## Author contributions

The author confirms being the sole contributor of this work and has approved it for publication.

## Footnotes

^1^The literature on 3 is immense. For a negative answer, see Dieks (2006).

^2^How local is “local” depends on the phenomena we want to describe.

^3^Information Gathering and Utilizing Systems (Hartle, 2005).

^4^Gruber (2008) is aware of this problem.

^5^See Mozersky (2011) for a clarification of this notion of existence.

^6^For Paul (2017, p. 262), however, the enduring and the perduring view of the self are compatible.

^7^Thanks to Carlo Rovelli for comments on this passage. See https://arxiv.org/abs/2007.05300.
